# Biotherapy in juvenile idiopathic arthritis Moroccan patients: a single-center experience

**DOI:** 10.11604/pamj.2022.41.135.27377

**Published:** 2022-02-16

**Authors:** Kenza Bouayed, Dalal Hamraoui, Nabiha Mikou, Asmaa Sakhi, Wassim Hilmi

**Affiliations:** 1 Department of Pediatric Rheumatology, Abderrahim Harouchi Children University Hospital, University Hospital Center Ibn Rochd, Casablanca, Morocco,; 2 Hassan II University of Medicine and Pharmacy, Casablanca, Morocco

**Keywords:** Juvenile idiopathic arthritis, biotherapy, tocilizumab, anakinra

## Abstract

In this study, we aimed to evaluate the clinicobiological findings and the biotherapy treatment response of Moroccan patients with juvenile idiopathic arthritis (JIA), and compare our results with those of populations of the same or different ethnicity. This retrospective cross-sectional study included patients aged 1-14 years, diagnosed between 2003 and 2018 with JIA according to the International League of Associations for Rheumatology (ILAR) 2004 revised criteria, who received biologics and who followed up during the year 2018 in the day hospital of our single-center tertiary pediatric rheumatology unit. Among 59 patients, 53% had systemic JIA, 29% seronegative polyarticular JIA, 8% arthritis-related enthesitis, 5% seropositive polyarticular JIA, 3% oligoarthritis and 2% psoriatic arthritis. Tocilizumab was the most prescribed biologic (34 patients), followed by Etanercept (25 patients), Adalimumab (6 patients), Anakinra (3 patients) and biosimilar Infliximab (3 patients). Eleven patients switched biologics. Erythrocyte sedimentation rate, number of active joints and the Juvenile Arthritis Disease Activity Score 27 (JADAS 27) decreased significantly at month three for 56 patients. These results were maintained at the last visit for 31 patients, while there was a slight worsening in 15 of them and no assessment in 13 patients due to lack of data. At the end of the evaluation, 39% of the patients were exclusively on biotherapy, while 61% were still on other disease-modifying antirheumatic drugs (DMARDs). Twenty-eight patients developed lymphopenia, 4 patients had elevated transaminases, 4 patients developed moderate infection, and 2 patients developed macrophage activation syndrome. To the best of our knowledge, this is the first Moroccan study on biotherapy in JIA. Our study population was characterized by a male predominance, a high frequency of the systemic form and a low percentage of positive antinuclear antibodies. We have shown that in the era of biologics, only 67.4% patients are nearly disease-free at the end of the study with a real risk of side effects. Although effective, biotherapy must be closely monitored because of potentially severe side effects, especially with Tocilizumab use.

## Introduction

Juvenile idiopathic arthritis (JIA) is the most common rheumatic disease in children, with a chronic clinical course and risk of impaired joint function. In recent decades, biotherapy has been shown to be effective and safe in JIA resistant to first-line disease-modifying antirheumatic drugs (DMARDs), and guidelines are now available for the use of biologic therapies in patients with JIA [1, 2]. According to these guidelines, it is recommended to start biologic therapy if disease activity remains moderate to high despite three months of methotrexate treatment. In our paediatric rheumatology department, we treat patients according to these guidelines whenever possible. However, in a developing country setting such as Morocco or sub-Saharan African countries, biotherapy has often been delayed due to its high cost and the major risk of infectious complications [3].

Generally, the use of biotherapy in our hospital depends on several other factors: first, the availability of treatment in the hospital, which cannot meet all the needs for the great demand; then the patient´s socio-economic status because only patients with health insurance or with a government-issued indigence card have access to this expensive treatment; and lastly the proximity of tertiary care structures, which are the only ones having access to costly treatments. Our objective is to share our own experience by presenting the clinical and laboratory results as well as treatment responses of JIA patients who were treated in our unit with biotherapy.

## Methods

**Study design and setting:** this is a retrospective cross-sectional study of 59 patients with JIA treated in the day hospital of our paediatric rheumatology tertiary care centre from January 1^st^ to December 31^st^, 2018. Patients were first diagnosed between 2003 and 2018 with JIA according to the ILAR 2004 revised criteria [4].

The JADAS 27 was selected as the disease activity measurement tool. It is based on four parameters: 1) physician´s global assessment of disease activity on a 0-10 visual analogue scale VAS; 2) patient´s/parent´s global disease assessment of well-being on a 0-10 VAS; 3) active joint numbers, evaluated in 27 joints; and 4) erythrocyte sedimentation rate (ESR) (normalized according to the 0-10 scale). The score is calculated by using the formula: physician VAS + patient/parent VAS + active joint count + ESR-20/10 (if the ESR<20, a score of 0 is given; if>120, a score of 10 is given).

Response to biotherapy was assessed on the basis of JADAS 27 before initiation of any biotherapy as a baseline, then at 3, 6, 12 months from initiation and at the last visit conducted in 2018. If the initial VAS was missing, it was considered as a maximum score of 10. If one or more parameters of JADAS 27 from the last visit in 2018 were missing or if the follow-up period was shorter than 6 months after the biotherapy initiation, the case was excluded from the therapeutic response section at the end of the study. Therefore, only 46 children could ultimately be fully evaluated. One of the limitations of our study is the use of JADAS 27 in the evaluation of ERA, activity, as it does not take into account axial involvement and enthesitis.

Inactive disease was considered as a state of no active arthritis, no systemic symptom, normal erythrocyte sedimentation rate (ESR) and JADAS 27 between 0-4. Mild disease was defined by a JADAS 27 between 5-10. Moderate disease activity was defined by a JADAS 27 between 11 and 20. No remission was obtained when the last JADAS 27 was greater than 20. JADAS 27 is not the most suitable tool for assessing the activity of the ERA because it does not take into account axial involvement or enthesitis.

**Study population:** the inclusion criteria were the age younger than 14 years at diagnosis, all subtypes of JIA, biotherapy treatment and a minimum follow-up time of 3 months since the start of biotherapy. Patients were excluded if they had other inflammatory, infectious or tumoural conditions that could mimic JIA. All patients were screened for latent or active tuberculosis before initiation of therapy, using chest X-rays, tuberculin skin test and a Quantiferon test. Two patients with positive Quantiferon were treated, prior to the biologics initiation, for latent tuberculosis using a 3-month course of isoniazid.

**Data collection:** we developed an operating form that included demographic data, JIA subtype, therapeutic management, clinical and biological treatment response parameters, and disease activity score. Data were collected from the patient's medical records.

**Statistical analysis:** statistical analysis was performed manually and controlled via excel. All files were analysed anonymously.

**Ethical considerations:** ethic approval was obtained from the local committee of A. Harouchi Mother and Child Hospital, CHU Ibn Rochd in Casablanca, Morocco.

## Results

**Epidemiological and clinical patients features:** thirteen of the 59 patients included could not be fully assessed at their last visit given, for ten of them, a post-biotherapy follow up shorter than 6 months, or because of missing one or more JADAS parameters for the remaining 3, making impossible the calculation of the JADAS. The gender distribution was almost equal, with a slight male predominance of 52.54%. Thirty-one patients (53%) had systemic JIA, seven (61.1%) had polyarticular JIA RF negative, three had polyarticular JIA RF positive, five (8%) had enthesitis related arthritis «ERA», two (3%) had oligoarticular JIA and the last one (2%) had psoriatic arthritis «PsA». The median age at diagnosis was six years with extremes of 1 and 14 years and the mean duration of follow up was 4 years and a half, ranging from 5 months to 15 years. Among the reported comorbidities, we counted three uveitis (with an ANA positive oligoarticular form, a seronegative polyarticular form and an ERA form), one cataract, 4 osteoporosis and 6 hip destructions.

**Laboratory findings:** anti-nuclear antibodies, rheumatoid factor, and HLA B27 were performed respectively in 53, 52, and 9 patients, and were positive respectively in 4/53 (7.54%), 3/52 (5.76%), and 2/9 (22.22%).

**Figure 1:**
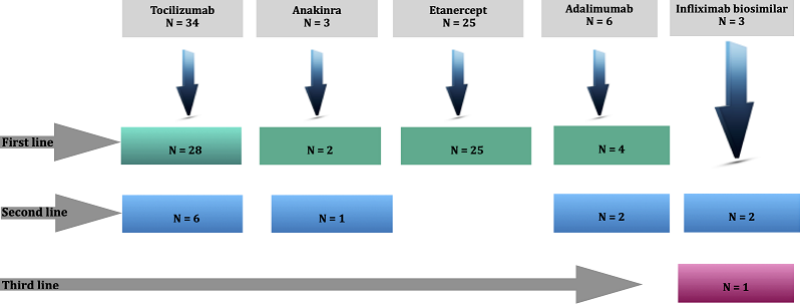
flow chart of biologics prescription

**Figure 2:**
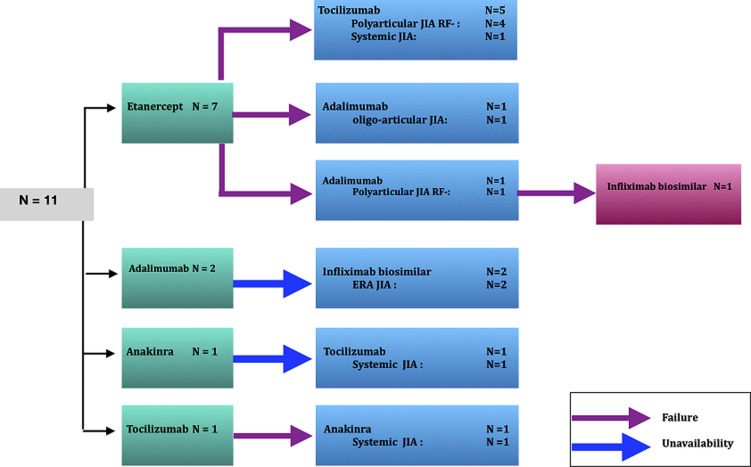
switch cases (N =11)

**Biological data:** the criteria for introducing biotherapy were as follows: a non-response of the peripheral joint forms to NSAIDs combined with methotrexate regularly taken for 3 months, a non-response of the axial forms to NSAIDs taken regularly for 3 months, and a non-response of the systemic forms to a 3-month treatment protocol combining NSAIDs and corticosteroids at a dose varying between 0.5 and 1 mg/kg/day, with or without methotrexate. Prior to biologics, all patients received corticosteroids, methotrexate or NSAIDs. Figure 1 illustrates the different biotherapies prescribed and their indications. Eleven patients (18.6%) received multiple biological agents, as explained in Figure 2.

The frequency of visits depended on the biological agent used. Patients were seen monthly or bi-monthly for Tocilizumab, depending on the indication; they were seen monthly for Etanercept and Adalimumab, and according to the infusion schedule for the Infliximab biosimilar. Patients on Anakinra were seen weekly because our hospital does not allow the delivery of more than one box of treatment supply per hospitalization. The patients were switched to a second or third line biotherapy agent if they did not respond or no longer responded to the prescribed biotherapy. Ineffectiveness was declared when the JADAS 27 was greater than 10 after 3 months of treatment with biotherapy. Lab work was performed monthly. It included CRP, ESR, Blood Cell Count, fibrinogen, transaminases, urea, creatinine. In this study, no patient was lost to follow-up because only the patients coming regularly to the day hospital during the year 2018 were included. The epidemiological and clinical characteristics of patients, as well as their laboratory findings and biologics data, are summarized in Table 1.

**Table 1:** clinical, laboratory characteristics and biologics data of JIA patients

Clinical data	N=59
**Gender**	28 Females/31 Males
**Age at diagnosis (years)**	
median (ages extremes)	6 (1-14)
**Duration of the follow-up (months)**	
median (ages extremes)	54 (5-180)
**JIA subtype n (%)**	
Systemic JIA	31 (53%)
Polyarticular JIA RF-	7 ( 29%)
Polyarticular JIA RF+	3 (5%)
ERA	5 (8%)
Oligoarticular JIA	2 (3%)
PsA	1 (2%)
**Laboratory findings**	n Positive/n Tested (%)
Anti-nuclear antibodies	4/53 (7.54%)
Rheumatoid factor	3/52 (5.76%)
HLA B27	2/9 (22.22%)
**Biologics data**	
**Age at initiation of biologics (years)**	
median (ages extremes)	7.73 (2-15)
**Initiation delay of biologics (%)**	
< 2 years	61%
> 2 years &< 4 years	27%
> 4 years	12%
**Associated treatments at biologics start**	%
Corticoids + NSAIDs + MTX	46%
NSAIDs + MTX	29%
Corticoids + NSAIDs	12%
Corticoids + MTX	7%
MTX	3%
Corticoids	3%
NSAIDs on request	2%
**Biologics type**	N=71
Tocilizumab	34
Anakinra	3
Etanercept	25
Adalimumab	6
Infliximab biosimilar	3
**Treatment at the last visit**	N=59
Biologics in mono therapy n (%)	23 (39%)
Biologics associated to other drugs n (%)	26 (61%)

JIA: Juvenile Idiopathic Arthritis, RF,: Rheumatoid Factor, ERA: Enthesitis Related to Arthritis, PsA: Psoriatic Arthritis, NSAIDs: Non Steroid Anti-Inflammatory Drugs, MTX: Methotrexate

The evolutive parameters on biologics are summarized in Table 2. At the end of the study, twenty-one patients had inactive disease, ten had mild activity disease, twelve had moderate activity disease and three had no remission. At the last visit, 39% were receiving biologics in monotherapy, while for the remaining 61%, other treatments could not be stopped. 56% of patients were maintained on the initial biological drugs, and 44% of them could benefit from an increased spacing of their biologics schedule. During follow up, side effects have been reported with a predominance of lymphopenia (Table 3). An active lymph node tuberculosis was diagnosed few years after the biologic initiation, which led to Tocilizumab temporary suspension and 6 months treatment with Isoniazid, Rifampicin and Pyrazinamide.

**Table 2:** evolutive parameters in patients with biotherapy

	At onset N=59	At 3 months N=59	At 6 months N=49	At 12 months N=40	At last visit N=46
ESR mm first hour	64.87	27.29	19.25	22.47	15.72
Active joint count	5.49	3.24	1.37	1.4	3.5
JADAS 27	26.97	10.10	6.88	5.75	6.21

ESR: Erythrocyte Sedimentation Rate, JADAS: Juvenile Arthritis Disease Activity Score

**Table 3:** adverse effects in patients with biotherapy

	Tocilizumab N=34	Anakinra N=3	Etanercept N=25	Adalimumab N=6	Infliximab bios N=3	Total AE N=45
Infections	4	0	0	0	0	4
LN TBK	1	0	0	0	0	1
Pneumonia	1	0	0	0	0	1
VHA	2	0	0	0	0	2
MAS	2	0	0	0	0	2
Anaphylaxia	0	1	0	0	0	1
Headeche/Dizziness	0	0	0	0	1	1
Pneumo-mediastin	1	0	0	0	0	1
Liver cytolysis related to biologics	2	1	0	0	1	4
Lymphopenia cell/μl	25	1	1	1	0	28
500-1000	17	0	0	0	0	17
1000-1500	8	1	1	1	0	11

LN TBK: lymph node tuberculosis, VHA: viral hepatitis A, MAS: macrophage activating syndrome, Infliximab bios: Infliximab biosimilar, AE: adverse events, MTX: methotrexate

## Discussion

JIA is the most common rheumatic disease in children. With the current improved understanding of its pathogenesis, biotherapy has become the gold standard in severe forms of the disease or those refractory to first-line therapy [5]. Various studies from different parts of the world showed differences in JIA characteristics including age of diagnosis, gender, and frequency of JIA subtypes. In our patients´ cohort, the mean age at diagnosis was six years (1-14) which is similar to a French study where the average age at diagnosis was 6.6 years, with extremes of 1 and 15 years [6]. Although studies from most regions report a female predominance, our series had the fourth-highest rate of male (52.5%), next to India (58.3%) [7], Turkey (53%) [8], and South Africa (50%) [9]. Concerning the disease distribution, the most frequent subtype was systemic JIA (53%) followed by polyarticular JIA RF- (29%). Our study highlights the relative paucity of the oligoarticular subtype compared to western countries [10]. The rarity of this subtype in Arab populations has already been documented in studies from Saudi Arabia and Oman [11, 12]. However, for us, this could also be a recruitment bias since these forms have a better prognosis, are not systematically referred to our tertiary care center, and require less biotherapy, which is one of our inclusion criteria.

Previous studies have shown differences in laboratory results, as illustrated in Table 4 [11, 13-15]. In our study population, ANA titres were much lower than published data, except in one South African study [9]. The goal of managing JIA is to induce disease remission and preserve daily functioning. Since biologic agents have been introduced, the prognosis has dramatically improved, but variable clinical responses have been observed depending on the selected biologic and the JIA subtype.

**Table 4:** laboratory findings in comparative studies

	Method	Period/data collection	Positive ANA n (%)	Positive RF n (%)	Positive HLA B27 n (%)
**Our Study N=59**	**Retrospective**	**1 year**	**4/53 (7.54%)**	**3/52 (5.76%)**	**2/9 (22.22%)**
Al-Hemairi MH 2016 Saudi Arabia N=82 [11]	Retrospective	7 years	30 (36.6%)	4 (4.9%)	1 (1.2%)
Danner S 2006 France N= 82 [13]	Retrospective	1 year	41 (61%)	2 (3%)	14 (21%)
Weakley K 2012 South Africa N=78 [9]	Prospective	1 year	3 (3.9%)	11 (14%)	18 (23%)
Abou EL Soud AM 2013 Egypt N: 132 [14]	Prospective	1 year	64 (48.5%)	36 (27.2%)	6 (4.5%)
Modesto C 2010 Spain N=432 15]	Retrospective and prospective	2 years	249 (57.6%)	3 (0.7%)	64 (14.8%)

ANA: anti-nuclear antibodies, RF: rheumatoid factor

In our study, biological agents were prescribed according to expert recommendations [16-27], whenever they were available in our hospital, and provided that the concerned patient had a government-issued indigent card or a health insurance. Although infliximab is not licensed for JIA because it failed to meet its primary endpoint in a randomized international study of patients with methotrexate (MTX) resistant polyarticular JIA [28], it is frequently used. CT-P13 is an infliximab biosimilar which is a less expensive but equally effective alternative; it has been approved for the same indications [29]. It is the only biosimilar available in our hospital, we have used it as a second or third line treatment, when etanercept or adalimumab were unavailable or have failed notably in patients living far from our hospital because of its spaced infusion interval. Of the 11 patients who switched due to failure or depletion of their original biologic at the hospital pharmacy, 10 were fully assessed and six (6/10=60%) had inactive or mild disease at the last examination. In a study including 1152 JIA patients, 270 (23%) started a second biologic, 61 (5%) started a third biologic, and 11 (1%) started a fourth biologic, with the achievement of a minimal disease activity that did not depend on the type of the switch molecule, making it impossible to orientate towards the switch biotherapy to be pro-posed [30].

As shown in other studies, we demonstrated that the active joint numbers, ESR and JADAS27 scores were significantly decreased at the third month of treatment for all JIA patients but 3 (56/59=95%). Almost all the assessable patients had inactive or mild disease at their last visit in 2018 (81.8%: 18/22 of patients on Tocilizumab; 46%: 6/13 of patients on Etanercept; 100%: one patient on Anakinra; and 60%: 6/10 of patients after a biologic switch).

In addition to the efficacy of Biologic disease-modifying anti-rheumatic drugs (bDMARDs), their safety has been evaluated and the most common adverse events are infections, skin reactions to the subcutaneous drug, cytopenia and transaminase elevation. We reported 4 infections with Tocilizumab, including previously unobserved lymph node tuberculosis, in contrast to the known risk of tuberculosis with anti-TNF [16, 31]. Consistent with previous studies where transaminase elevation is common with all biotherapy agents, especially with concomitant use of MTX [19], we reported hypertransaminasemia in 4 patients. Instead of the neutropenia usually reported with tocilizumab, 25 of our Tocilizumab patients developed lymphopenia [32]. In addition, we report MAS in two patients on Tocilizumab, as previously described, without causal relationship [32, 33].

## Conclusion

To the best of our knowledge, it is the first Moroccan study evaluating biotherapy in JIA. Our study population is characterized by a male predominance, a high frequency of the systemic form and a low percentage of positive anti-nuclear antibodies. We showed that biotherapy is effective, especially Tocilizumab, but with a significant risk of side effects requiring regular patient monitoring. A prospective study is needed to confirm our results.

### What is known about this topic

- Biotherapy showed its efficacy in JIA patients with a good short and long term tolerance;
- While mild adverse reactions are often reported, serious adverse events rarely happen. Their incidence may decline with patient's proper monitoring, temporary discontinuation of biotherapy when needed, and treatment of the incident as required.

### What this study adds

- To the best of our knowledge, it is the first Moroccan study about biotherapy in JIA;
- Our study showed a difference in the patients´ characteristics, with a male predominance, a rarity of the oligoarticular subtype, and a rare percentage of positive anti-nuclear antibodies;
- We showed that biotherapy is effective in JIA patients, specifically Tocilizumab, but with a real risk of side effects requiring regular monitoring of patients on biotherapy.

## References

[1] Ringold S, Angeles-Han ST, Beukelman T, Lovell D, Cuello CA, Becker ML (2019). 2019 American College of Rheumatology/Arthritis Foundation Guideline for the Treatment of Juvenile Idiopathic Arthritis: Therapeutic Approaches for Non-Systemic Polyarthritis. Sacroiliitis and Enthesitis. Arthritis Care Res (Hoboken).

[2] Ringold S, Weiss PF, Beukelman T, DeWitt EM, Ilowite NT, Kimura Y (2013). 2013 update of the 2011 American College of Rheumatology recommendations for the treatment of juvenile idiopathic arthritis: recommendations for the medical therapy of children with systemic juvenile idiopathic arthritis and tuberculosis screening among children receiving biologic medications. Arthritis Rheum.

[3] Scott C, Webb K (2014). Paediatric rheumatology in sub-Saharan Africa. Rheumatology (Oxford).

[4] Petty RE, Southwood TR, Manners P, Baum J, Glass DN, Goldenberg J (2004). International League of Associations for Rheumatology. International League of Associations for Rheumatology Classification of Juvenile Idiopathic Arthritis: Second Revision Edmonton, 2001. J Rheumatol.

[5] Stoll ML, Gotte AC (2008). Biological therapies for the treatment of juvenile idiopathic arthritis: Lessons from the adult and pediatric experiences. Biologics.

[6] Solau-Gervais E, Robin C, Gambert C, Troller S, Danner S, Gombert B (2010). Prevalence and distribution of juvenile idiopathic arthritis in a region of Western France. Joint Bone Spine.

[7] Kunjir V, Venugopalan A, Chopra A (2010). Profile of indian patients with juvenile onset chronic inflammatory joint disease using the ILAR classification criteria for JIA: a community-based cohort study. J Rheumatol.

[8] Yilmaz M, Kendirli G, Altintas DU, Karakoc GB, Inal A, Kilic M (2008). Juvenile idiopathic arthritis profile in turkish children. Pediatr Int.

[9] Weakley K, Esser M, Scott C (2012). Juvenile idiopathic arthritis in two tertiary centres in the Western Cape, South Africa. Pediatric Rheumatology Online J.

[10] Berthold E, Maånsson B, Kahn R (2019). Outcome in juvenile idiopathic arthritis: a population-based study from Sweden. Arthritis Res Ther.

[11] Al-Hemairi MH, Albokhari SM, Muzaffer MA (2016). The Pattern of Juvenile Idiopathic Arthritis in a Single Tertiary Center in Saudi Arabia. Int J Inflam.

[12] Abdwani R, Abdalla E, Al Abrawi S, Al-Zakwani I (2015). Epidemiology of juvenile idiopathic arthritis in Oman. Pediatr Rheumatol Online J.

[13] Danner S, Sordet C, Terzic J, Donato L, Velten M, Fischbach M (2006). Epidemiology of juvenile idiopathic arthritis in Alsace, France. J Rheumatol.

[14] Abou El-Soud AM, El-Najjar AR, El-Shahawy EE, Amar HA, Hassan TH, Abd-Allaha SH (2013). Prevalence of juvenile idiopathic arthritis in Sharkia Governorate, Egypt: epidemiological study. Rheumatol Int.

[15] Modesto C, Antón J, Rodriguez B, Bou R, Arnal C, Ros J (2010). Incidence and prevalence of juvenile idiopathic arthritis in Catalonia (Spain). Scand J Rheumatol.

[16] Cimaz R, Maioli G, Calabrese G (2020). Current and emerging biologics for the treatment of juvenile idiopathic arthritis. Expert Opin Biol Ther.

[17] De Benedetti F, Brunner HI, Ruperto N (2012). PRINTO; PRCSG. Randomized trial of tocili-zumab in systemic juvenile idiopathic arthritis. N Engl J Med.

[18] Yokota S, Imagawa T, Mori M, Miyamae T, Aihara Y, Takei S (2008). Efficacy and safety of tocilizumab in patients with systemic-onset juvenile idiopathic arthritis: a randomised, double-blind, placebo-controlled, withdrawal phase III trial. Lancet.

[19] Brunner HI, Ruperto N, Zuber Z, Keane C, Harari O, Kenwright A (2015). Efficacy and safety of tocilizumab in patients with polyarticular-course juvenile idiopathic arthritis: results from a phase 3, randomised, double-blind withdrawal trial. Ann Rheum Dis.

[20] Ter Haar NM, Dijkhuizen EHP, Swart JF, van Royen-Kerkhof A, El Idrissi A, Leek AP (2019). Treatment to target using recombinant interleu-kin-1 receptor antagonist as first-line monotherapy in new-onset systemic juvenile idiopathic arthritis: results from a five-year follow-up study. Arthritis Rheumatol.

[21] Lovell DJ, Giannini EH, Reiff A, Cawkwell GD, Silverman ED, Nocton JJ (2000). Etanercept in children with polyarticular juvenile rheumatoid arthritis. Pediatric Rheumatology Collaborative Study Group. N Engl J Med.

[22] Shenoi S, Wallace CA (2010). Tumor necrosis factor inhibitors in the management of juvenile idiopathic arthritis: an evidence-based review. Paediatr Drugs.

[23] Horneff G, Schmeling H, Biedermann T, Foeldvari I, Ganser G, Girschick HJ (2004). The German etanercept registry for treatment of juvenile idiopathic arthritis. Ann Rheum Dis.

[24] Giannini EH, Ilowite NT, Lovell DJ, Wallace CA, Rabinovich CE, Reiff A (2009). Long-term safety and effectiveness of etanercept in children with selected categories of juvenile idiopathic arthritis. Arthritis Rheum.

[25] Horneff G, Foeldvari I, Minden K, Trauzeddel R, Kümmerle-Deschner JB, Tenbrock K (2015). Efficacy and safety of etanercept in patients with the enthesitis-related arthritis category of juvenile idiopathic arthritis: results from a phase III random-ized, double-blind study. Arthritis Rheumatol.

[26] Lovell DJ, Ruperto N, Goodman S, Reiff A, Jung L, Jarosova K (2008). Adalimumab with or without methotrexate in juvenile rheumatoid arthritis. N Engl J Med.

[27] Hugle B, Burgos-Vargas R, Inman RD, O'Shea F, Laxer RM, Stimec J (2014). Long-term outcome of anti-tumour necrosis factor alpha blockade in the treatment of juvenile spondyloarthritis. Clin Exp Rheumatol.

[28] Ruperto N, Lovell DJ, Cuttica R, Wilkinson N, Woo P, Espada G (2007). A randomized, placebo-controlled trial of infliximab plus methotrexate for the treatment of polyarticular-course juvenile rheumatoid arthritis. Arthritis Rheum.

[29] Gabbani T, Deiana S, Annese V (2017). CT-P13: design, development, and place in therapy. Drug Des Devel Ther.

[30] Kearsley-Fleet L, Heaf E, Davies R, Baildam E, Beresford MW, Foster HE (2020). Frequency of biologic switching and the outcomes of switching in children and young people with juvenile idiopathic arthritis: a national cohort study. Lancet Rheumatol.

[31] Machado SH, Xavier RM (2017). Safety of tocilizumab in the treatment of juvenile idiopathic arthritis. Expert Opin Drug Saf.

[32] Klein A, Klotsche J, Hügle B, Minden K, Hospach A, Weller-Heinemann F (2020). Long-term surveillance of biologic therapies in systemic-onset juvenile idiopathic arthritis: data from the German BIKER registry. Rheumatology (Oxford).

[33] De Benedetti F, Schneider R, Weitzman S, Devlin C, Daimaru K, Yokota S (2014). Macrophage activation syndrome in patients with systemic juvenile idiopathic arthritis treated with tocilizumab. Pediatr Rheumatol Online J.

